# Is the C-terminal insertional signal in Gram-negative bacterial outer membrane proteins species-specific or not?

**DOI:** 10.1186/1471-2164-13-510

**Published:** 2012-09-26

**Authors:** Nagarajan Paramasivam, Michael Habeck, Dirk Linke

**Affiliations:** 1 Department I, Protein Evolution, Max Planck Institute for Developmental Biology, Tübingen, Germany

**Keywords:** Outer membrane β-barrel protein biogenesis, Clustering, Hellinger distance, CLANS, Species specificity, Short linear motifs, GLAM2, C-terminal β-strand, BamA, β-barrel assembly machinery, Gram-negative bacteria, Outer membrane, Principal component analysis, Frequency plots

## Abstract

**Background:**

In Gram-negative bacteria, the outer membrane is composed of an asymmetric lipid bilayer of phopspholipids and lipopolysaccharides, and the transmembrane proteins that reside in this membrane are almost exclusively β-barrel proteins. These proteins are inserted into the membrane by a highly conserved and essential machinery, the BAM complex. It recognizes its substrates, unfolded outer membrane proteins (OMPs), through a C-terminal motif that has been speculated to be species-specific, based on theoretical and experimental results from only two species, *Escherichia coli* and *Neisseria meningitidis*, where it was shown on the basis of individual sequences and motifs that OMPs from the one cannot easily be over expressed in the other, unless the C-terminal motif was adapted. In order to determine whether this species specificity is a general phenomenon, we undertook a large-scale bioinformatics study on all predicted OMPs from 437 fully sequenced proteobacterial strains.

**Results:**

We were able to verify the incompatibility reported between *Escherichia coli* and *Neisseria meningitidis*, using clustering techniques based on the pairwise Hellinger distance between sequence spaces for the C-terminal motifs of individual organisms. We noticed that the amino acid position reported to be responsible for this incompatibility between *Escherichia coli* and *Neisseria meningitidis* does not play a major role for determining species specificity of OMP recognition by the BAM complex. Instead, we found that the signal is more diffuse, and that for most organism pairs, the difference between the signals is hard to detect. Notable exceptions are the *Neisseriales*, and *Helicobacter spp*. For both of these organism groups, we describe the specific sequence requirements that are at the basis of the observed difference.

**Conclusions:**

Based on the finding that the differences between the recognition motifs of almost all organisms are small, we assume that heterologous overexpression of almost all OMPs should be feasible in *E. coli* and other Gram-negative bacterial model organisms. This is relevant especially for biotechnology applications, where recombinant OMPs are used e.g. for the development of vaccines. For the species in which the motif is significantly different, we identify the residues mainly responsible for this difference that can now be changed in heterologous expression experiments to yield functional proteins.

## Background

In Gram-negative bacteria, the cytoplasm is surrounded by inner membrane (IM) and outer membrane (OM), which are separated by an inter-membrane space, called the periplasm. Most of the newly synthesized proteome remains in the cytoplasm, but in addition, different machineries are involved in the translocation of non-cytoplasmic proteins to different subcellular localizations, including the inner or outer membrane, the periplasmic space, or the extracellular space. Some of these machineries recognize their substrate proteins by an N-terminal signal peptide (SP) for the translocation process, while other machineries are SP-independent. The IM, which is a phospholipid lipid bilayer, is mostly occupied by transmembrane α-helical proteins, by inner membrane lipoproteins on its periplasmic side, and by other membrane associated proteins on both sides of the membrane. In contrast, the asymmetric OM, which consists of phospholipids only in the inner leaflet of the membrane and lipopolysaccharides in the outer leaflet, is mostly occupied by transmembrane (outer membrane) β-barrel proteins, and by outer membrane lipoproteins on its periplasmic side [1].

The biogenesis of an outer membrane β-barrel protein (OMP) begins with the translocation of the newly synthesized, unfolded protein across the IM into the periplasm via the Sec translocation machinery, which requires a cleavable general SP. Once the unfolded OMP reaches the periplasm, it uses the SurA or Skp-DegP pathway to reach the OM. SurA, Skp and DegP are periplasmic chaperones, which interact with unfolded OMPs by protecting them from aggregation and thus help them to reach the OM [2,3]. It has been shown that the SurA pathway and the Skp/DegP pathway can work in parallel, but that the SurA pathway plays an important role when the cell is under normal growth conditions, while under stress conditions, the Skp-DegP pathway plays the major role [4,5].

Once periplasmic chaperones deliver the OMPs to the OM, the folding and insertion of the protein into the membrane is mediated by the β-barrel assembly machinery (BAM), without an external energy source [6] such as ATP or ion gradients. This machinery involves an essential multi-domain protein, BamA (Omp85), which consists of a 16-stranded transmembrane β-barrel domain, and of a large periplasmic part that consists of five POTRA (polypeptide transport-associated) domains. BamA is highly conserved in Gram-negative bacteria and also has homologues in mitochondria (Sam50) and chloroplasts (Toc75-V) [2]. In addition, the BAM complex, at least in *E. coli*, consists of four lipoproteins, BamB, BamC, BamD and BamE, among which only BamD is essential and conserved in most Gram-negative bacteria [2]. Recent HMM-based sequence analysis by Anwari *et al. *[7] showed that BamB and BamE are mainly present in α-, β- and γ-proteobacteria, while BamC is present only in β- and γ-proteobacteria. They also found a new lipoprotein subunit in the BAM complex, named BamF, which is present exclusively in α-proteobacteria.The BAM complex recognizes OMPs as its substrates via binding to an amphipathic C-terminal β-strand of the unfolded β-barrel [8], but the exact binding mode is still not clear. It was suggested that C-terminal β-strand binds to BamD [9], once the unfolded OMPs are delivered to the BAM complex by periplasmic chaperones. But a recent BamC and BamD subcomplex crystal structure shows that the unstructured N-terminus of BamC binds to the proposed substrate binding site of BamD [4]. The C-terminal β-strand of an OMP β-barrel domain typically contains an aromatic residue at its C-terminus. It has been reported that deletion or substitution of this C-terminal residue negatively affects the biogenesis of OMPs [10,11]. Also, *in vitro* studies showed that the *E. coli* OM porin PhoE, when lacking its C-terminal Phe residue, fails to open the Omp85/BamA channel [8]. In both studies, overexpression of the mutant OMP was lethal to the cells. At lower concentration, the mutant protein was tolerated and got inserted into the membrane. This leads to the suggestion that a weak insertion signal other than the C-terminal residue or β-strand is present [8].

Robert *et al.*[8] observed that the *N. meningitidis* OM porin PorA or its C-terminal β-strand did not open the *E. coli* Omp85/BamA channel, and the comparison of the C-terminal β-strands from *N. meningitidis* and *E. coli* OMPs showed a high preference of positive amino acids at the penultimate (+2) position in neisserial OMPs. When they mutated *E. coli* PhoE or its C-terminal β-strand, changing Gln for Lys at the +2 position, it did not open the channel any more; in contrast, a *Neisseria* PorA peptide with Gln instead of Lys increased the channel activity considerably. These studies and the fact that high concentrations of neisserial OMPs were lethal in *E. coli* cells, lead to the conclusion that the C-terminal insertion signal is species-specific and that the residues at the +2 position were important for this phenomenon. The number of peptides/proteins used in the comparison in the study [8] was very low, compared to the total number of OMPs present in the *E. coli* or *N. meningitidis* genomes; moreover, the phenomenon was only compared between two organisms, one β- and one γ-proteobacterial species. Since neisserial OMPs could be expressed in *E. coli* at low expression rates, either the neisserial C-terminal insertion signal is weakly recognized by *E. coli* BAM complex, or other β-strands in the full length protein might act as a weak insertion signal.

Thus, there seems to be at least some overlap in the peptide recognition. The intention of this study was to use computational methods to quantify this overlap, and to find out whether the observed (partial) species specificity of the insertion signal is exhibited by all Gram-negative bacterial organisms.

## Results and discussion

We identified 22,447 OMPs from 437 Gram-negative bacteria using PSORTb [12], CELLO [13] and HHomp [14] as described in the methods section. These OMPs can be classified into different outer membrane protein (OMP) classes/families based on their function and the number of β-strands present in them, as these two features are usually coupled [14-17]. We used HHomp [14] to classify the proteins into different OMP families. A brief summary of the OMP classification obtained from HHomp [14] for our data set is shown in Table 1. We then used ProfTMB [18] and PSIPRED [19] annotations to identify and extract the C-terminal β-strands from the OMPs. To evaluate the phenomenon of species specificity, we initially tried to cluster the C-terminal β-strands using different methods, such as sequence based clustering in CLANS [20] and organism-specific PSSM profile-based hierarchical clustering. Since the sequences were highly similar and very short, the results obtained from these methods were not helpful to our analysis. We then used chemical descriptors and represented each amino acid in the peptides by five-dimensional vectors, thus representing each 10-residue peptide as a 50-dimensional vector. Next, we used dimensionality reduction techniques (principal component analysis) to reduce the dimensions to 12 (the lowest number of dimensions that still contains most of the difference information, see Methods). We then used all peptide vectors from an organism to derive a multivariate Gaussian distribution, which we describe as the ‘peptide sequence space’ of the organism. The overlap between these multidimensional peptide sequence spaces (multivariate Gaussian distributions) was calculated using a statistical theory method, the Hellinger distance. As described in the methods section, the pairwise overlaps between organism sequence spaces were used to cluster them in CLANS [20].

**Table 1:** Dataset classified based on OMP class

**OMP class**	**# of β-strands**	**Total # of peptides**	**OMP class found in # of organisms in different proteobacteria class**					**Function/Protein family**
			**α**	**β**	**γ**	**δ**	**ɛ**	
OMP.8	8	2300	71	77	227	24	10	Membrane anchors [15]
OMP.10	10	95	5	2	66	2	2	Bacterial proteases [16]
OMP.12	12	1550	60	75	212	18	10	Integral membrane enzymes [15]
OMP.14	14	572	47	38	221	20	22	Long chain fatty acid transporter [17]
OMP.16	16	2477	41	86	210	23	8	General porins [15]
OMP.18	18	327	2	14	134	7	1	Substrate specific porins [15]
OMP.22	22	7462	71	86	231	25	23	TonB-dependent receptors [15]
OMP.nn	Not known	7591	71	86	231	26	23	-
OMP.hypo	Not known	73	2	18	33	9	1	-

The OMP class of a protein was predicted by HHomp [14]. HHOmp defines the class based on the similarity to a closely related OMP structure. When HHomp cannot find a related structure, it classifies the proteins in OMP.nn. OMP.hypo proteins are hypothetical proteins [14].

### Clustering of organisms based on C-terminal β-strands

The pairwise comparison of the overlap between sequence spaces should help us to predict the similarity between the C-terminal insertion signal peptides, and how high the probability is that the protein of one organism can be recognized by the insertion machinery of another organism. When there is a complete overlap of sequence space between two organisms, we assume that all C-terminal insertion signals from one organism will be recognized and functionally expressed by another organism’s BAM complex and vice-versa. When there is only little overlap between the sequence spaces of two organisms, we assume that only a small number of C-terminal insertion signals from one organism will be recognized by another organism’s BAM complex. When there is no overlap, we assume that there is a general incompatibility.

**Figure 1:**
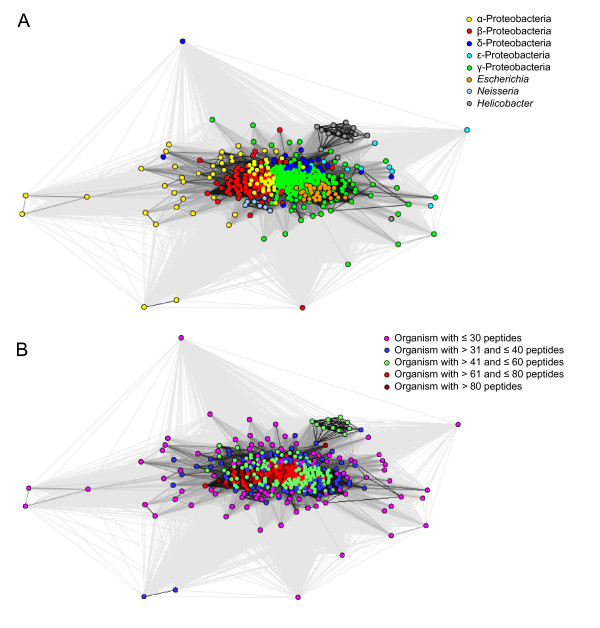
**Cluster map based on 437 sequenced Gram-negative organisms.** In the cluster map each node represents one organism. The Hellinger distance was used to calculate the pairwise overlap between the multi-dimensional peptide sequence spaces of organisms. The calculated similarity or overlap was used to cluster the organism in CLANS. Figure 1**A** is colored by taxonomic class and Figure 1**B** is colored by the number of peptides in each organism.

As described in the methods section, we examined the overlap of peptide sequence spaces between 437 Gram-negative bacterial organisms and used the pairwise overlap measurement to cluster the organisms. Since the C-terminal β-strands are highly conserved between all OMPs [21], it was very difficult to select a particular cut-off for the distance measure. Thus, the clustering was carried out using all the distance measures obtained from the calculations. In the resulting 2D cluster map (Figure 1A), each node is one out of the 437 organisms, and they are colored based on the taxonomic classes (see the figure legend). During clustering with default clustering parameters in CLANS [20], the organisms tended to collapse into a single point, which illustrates that there is large overlap between the peptide sequence spaces. Thus, we introduced very high repulsion values and minimum attraction values in CLANS [20] during clustering. With these settings the organisms formed a central big cluster, but separated crudely according to their taxonomic classes. We repeated the clustering multiple times to ensure that this separation is reproducible. In the cluster map (Figure 1A), β- and γ-Proteobacteria form two sub-clusters, separated by the α-Proteobacteria. The very few δ-Proteobacteria in our data set cluster in the periphery of the γ-proteobacterial cluster. In the cluster map, *E. coli* strains cluster along with other γ-Proteobacteria. Even though *Neisseria* species cluster along with other β-Proteobacteria, they form a sub-cluster and are found in the periphery of the β-proteobacterial cluster. Note also that in this map, *Helicobacter* species form a distinct cluster well separated from the rest of the organisms. This core cluster includes *H. pylori* strains, *H. acinonychis* and *H. felis,* but not *H. hepaticus* and *H. mustelae* species. The remaining ε-proteobacteria species are scattered in the periphery of the cluster map. The distinct cluster formed by most *Helicobacter* species demonstrates that the sequence spaces of *Helicobacter* species are significantly different from rest of the organisms. The neisserial cluster had only very few strong connections even with other β-proteobacterial organisms, which means the overlap or similarity of peptide sequence space between *Neisseriales* with rest of the β-Proteobacteria is comparatively low. When we used stringent thresholds for the distance measure, we noticed that the *Neisseria* and *Helicobacter* clusters started to move even further away from the center of the cluster map.

### Control experiments for clustering: randomly shuffled peptide sequences lose the signal for clustering

We noticed that the organisms seen at the periphery of the cluster map had a lower overall number of peptides, while organisms with more peptides are typically seen at the center of the circle. The cluster map in Figure 1B is colored based on the number of extracted peptides per organism. In Figure 1B, there are 99 organisms which have ≤ 30 peptides (colored in pink), 77 organisms with 31 to 40 peptides (colored in blue), 136 organisms with 41 to 60 peptides (colored in green), 66 organisms with 61 to 80 peptides (colored in red), and 59 organisms with more than 80 peptides (colored in brown). Even though *H. pylori* strains have a comparably high number of peptides (43 to 51 peptides), they still form a separate cluster in the periphery of the cluster map; therefore there must be an underlying organism-specific signal from the contributing peptides at least in this case.

**Figure 2:**
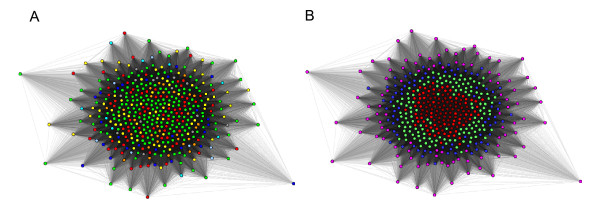
**CLANS cluster map of randomly shuffled peptides from 437 organisms.** Figure 2**A** is colored by taxonomic class and Figure 2**B** is colored by the number of peptides in an organism. Colors are similar to Figure 1.

To confirm the presence of the organism-specific signal, we took peptides from all the organisms and shuffled the positions of their amino acids randomly, and derived a new similarity matrix as mentioned in the method section which we clustered in CLANS [20]. Figure 2A shows the results from this test, where one can notice the taxonomic specific separations were completely lost. The cluster map in Figure 2B, colored based on the abundance of OMPs in an organism, shows that organisms with more peptides are in the center, and organisms with fewer peptides move to the outer rim of the cluster map. This test confirms that the there is a species-specific signal for which the position of the individual amino acids is important; this is lost when the residues in the peptides are shuffled randomly.

### High preference of positively charged residues at the +2 position in *Neisseria* species

**Figure 3:**
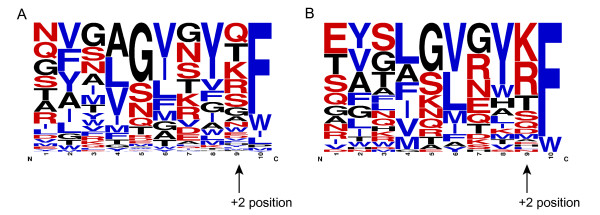
**Frequency plots derived from unique C-terminal insertion signal peptides for *Escherichia *(Figure 3A) and *Neisseria *(Figure 3B) strains.** Frequency plots were made from 188 unique peptides of 31 *Escherichia* strains and 50 unique peptides of 7 *Neisseria* strains. The +2 position is indicated by the arrow in the figure. *Escherichia* strains (Figure 3A) have no strong preference for any amino acid at the +2 position, whereas *Neisseria* strains (Figure 3B) have a strong preference for positively charged amino acids (Arg and Lys) at the +2 position. Hydrophobic residues are colored in blue and polar residues are colored in red.

**Figure 4:**
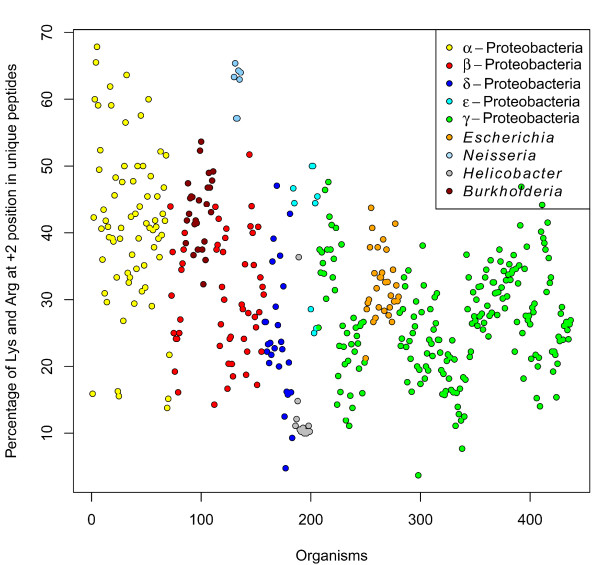
**Percentage of Arg and Lys at +2 positions.** We calculated the percentage of Arg and Lys residues at the +2 position from all unique peptides from the 437 organisms; color is based on taxonomic class. The *Neisseria* strains show a high preference for positively charged amino acids at the +2 position compared to other organisms.

The comparison of the C-terminal peptide sequences in the β-barrel of selected OMPs of *E. coli* and *N. meningitidis* peptides by Robert *el al *[8] showed a strong preference for positively charged amino acids (Arg and Lys) at the +2 position in neisserial OMPs, which led to the suggestion of a distinct species specificity of the C-terminal β-strand recognition. Since the comparison was made from 11 and 9 OMPs from *E. coli* and *N.meningitidis,* respectively, we wanted to confirm this with a larger set of OMPs from the same bacterial species. The frequency plots in Figure 3A and B were created from 171 (*E. coli*) and 50 (*N.meningitidis*) unique C-terminal β-strands. Comparison between these plots demonstrates the high preference of Arg and Lys at the +2 position in neisserial OMPs. When we checked the frequency of amino acids at the +2 position for 22,447 peptides from all 437 organisms, we noticed that in the complete dataset, Arg and Lys are the top two preferred residues at the +2 position, and that they are present in 31.62% (3996 + 3102) of the peptides. A similar frequency of Arg and Lys (31.32% (2262 + 1794 out of 12,949 unique peptides)) is observed when only taking unique peptides into account (i.e. when duplicates are removed from the database). Figure 4 shows the percentage of Arg and Lys at the +2 position in 437 organisms; in this plot, *Neisseria* strains stand apart even from other β-proteobacterial organisms, and also from all other proteobacterial organisms. *Neisseria* strains (and a few α-proteobacterial organisms) have more than 60% of peptides with positively charged residues at the +2 position. Note, though, that also in all other organisms, positive charges are abundant there; for example, different *Escherichia* strains also have 25-40% of peptides with Arg and Lys at the +2 position. Thus, when these proteins are expressed, the *Escherichia* BAM complex should be able to recognize proteins with positively charged residues at +2 positions. As a matter of fact, there is experimental evidence for the functional expression of OMPs with positively charged residues at the +2 position in *E. coli *[22].

### High preference of Histidine at the +3 position in porins (16-stranded OMPs) from β-proteobacteria

**Figure 5:**
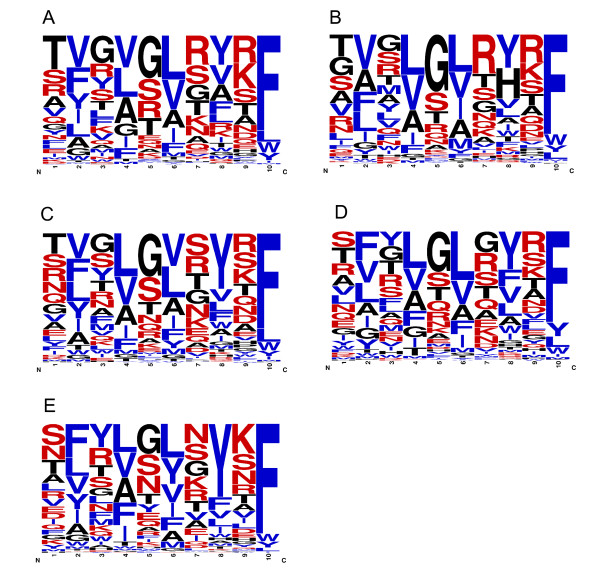
**Frequency plots of C-terminal β-strands from Proteobacteria.** Frequency plots generated from unique peptides of α-proteobacteria are shown in Figure 5**A**, of β-Proteobacteria in Figure 5**B**, of γ-Proteobacteria in Figure 5**C**, of δ-Proteobacteria in Figure 5**D** and of ε-Proteobacteria in Figure 5**E**. The frequency plots are overall very similar; an exception is the high frequency of His at the +3 position in β-Proteobacteria and of Tyr at the +5 position in ε-Proteobacteria.

**Figure 6:**
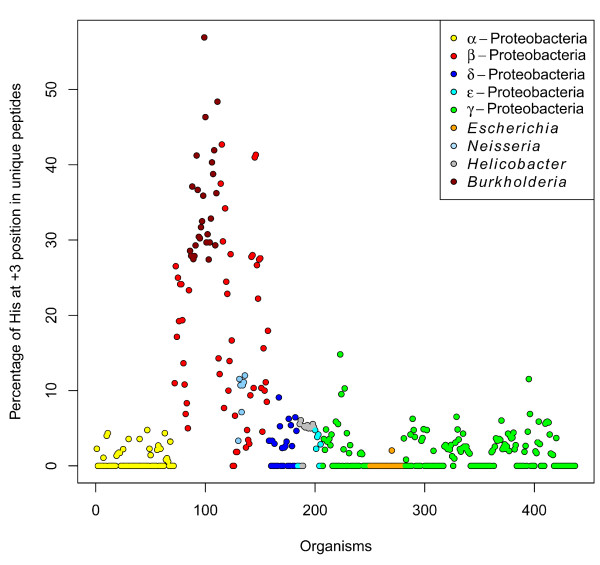
**Frequency of His at the +3 position.** The percentage of His at +3 was calculated from all unique peptides from 437 organisms. A high preference for His at +3 is observed for 16-stranded OMPs of β-Proteobacteria. Since there is a high number of 16-stranded OMPs in *Burkholderia* strains (see Additional file 1 and Additional file 2), they were also annotated in the plot.

In the frequency plots (Figure 5) generated for each taxonomic class of Proteobacteria, we observed that the frequency of amino acids in the +2 positions were comparable, with the possible exception of the *Neisseriae*. In contrast to that, we observed a prevalence (up to 57% frequency) of His at the +3 position for β-proteobacteria, while the other taxonomic classes shared a similar, low (<15%) frequency of His in that position (Figure 6). 80% of the peptides with His at the +3 position belong to the β-proteobacteria and more than 92% of these peptides stem from 16-stranded β-barrel proteins (Porins, denoted as the OMP.16 class by HHOmp). None of the *Escherichia* C-terminal β-strands in our database have His at the +3 position, and experiments by Robert *et al.* were done with a *Neisseria* PorA peptide with a His at the +3 position. This might be the true reason why *E. coli* BamA didn’t recognize neisserial peptides. When we further examined the available structures of porins from *Neisseria*, and we found the His at the +3 position to be present in the trimerization interface of the porins. Since the vast majority of the His residues at the +3 position of the C-terminal motifs were from 16-stranded porins that typically trimerize, this position might be relevant for trimerization in neisserial porins.

### High preference of Tyrosine at the +5 position in *Helicobacter* species

**Figure 7:**
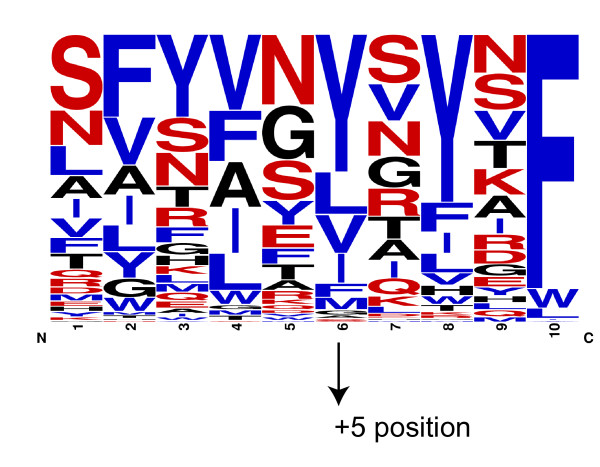
**Frequency plot of unique C-terminal β-strands from *Helicobacter *species.** 163 unique C-terminal insertion signals from 14 *Helicobacter* strains were used to generate this plot. The +5 position which has the strong preference of Tyr is marked with the arrow.

**Figure 8:**
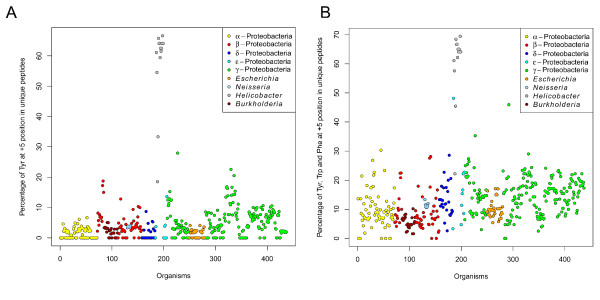
**The percentage of Tyr (Figure 8A) and aromatic hydrophobic amino acids (Figure 8B) at the +5 position.** For Figure 8A, we calculated the percentage of Tyr at the +5 position from all unique peptides from 437 organisms and for Figure 8B, we calculated the frequency of Tyr, Phe and Trp at the +5 position from all unique peptides from 437 organisms. In both plots *Helicobacter* strains shows a high preference of Tyr and aromatic amino acids at the +5 position.

The separate cluster formed by *Helicobacter* species was an interesting observation for us, because it forms a more distinct cluster than *Neisseria*. This means that the peptide sequence space of *Helicobacter* species is more different from the rest of the organisms than even the one of *Neisseriales*. But the frequency plots (Figure 7A and B), generated from unique peptides of all *Helicobacter* species and *H. pylori* strains respectively, did not show a strong preference for any amino acid at either the +2 position and the strong preference of Tyr at +3 position is common among the c-terminal insertion signals. But, we noticed an uncommon strong preference of Tyr at the +5 position. The presence of a hydrophobic residue is common at +5 positions, but the presence of aromatic hydrophobic amino acids (especially Tyr) at the +5 position are highly preferred in *H. pylori* strains compared to other organisms (Figure 8A and B). Since the peptide sequence space depends upon the entire sequence, we cannot confirm that the separate cluster formed by the *H. pylori* is exclusively due to the residues at this one particular position. There is experimental evidence that the expression of various *H. pylori* OMPs in *E. coli* is problematic [23]. Fisher *et al.* noticed that as long as the expressed *H. pylori* OMP remains in the cytoplasm of *E. coli*, it is not lethal, but that once it is secreted to the periplasm by the Sec machinery, it becomes lethal to *E. coli*. They also mentioned - without showing data - that removal of the C-terminal β-barrel region resulted in toleration of the proteins in the periplasmic space. This probably means that the *E. coli* BAM complex didn’t recognize the C-terminal β-strands of the *H. pylori* OMPs, and the subsequent aggregation of the OMPs in the periplasm and the blockage of the BAM complex lead to the lethality. The authors concluded that the difference in OM lipid composition of *Helicobacter*, which contains cholesteryl glycosides [24], might have imposed some structural constraints on the OMP structure, and that this structural change is not tolerated by other organisms resulting in the observed lethality of such constructs.

### OMP class-specific and taxonomy class-specific signals

**Figure 9:**
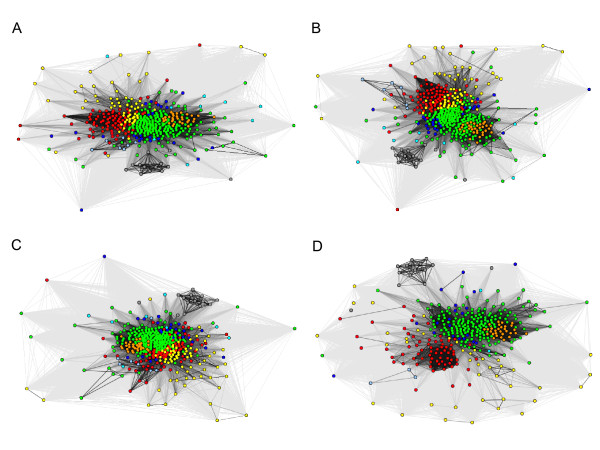
**Control experiments to show the influence of overrepresented OMP classes.** OMP classes OMP.8 (Figure 9**A**), OMP.12 (Figure 9**B**), OMP.16 (Figure 9**C**) and OMP.22 (Figure 9**D**) were removed and only organisms with more than 20 unique peptides were used in the clustering. Peptides belonging to OMP.nn and OMP.hypo (OMPs with unknown strand number and function) were not removed from the data set during the control experiments. Color legends are similar to the Figure 1A.

We noticed that in some organisms, certain OMP classes of proteins are over-represented (see figure in Additional file 1). Examples are the prevalence of 16-stranded β-barrels in the genomes of some β-proteobacteria and 22-stranded β-barrels in the genomes of some α-proteobacteria (see Additional file 2). Moreover, of the 22,447 sequences in the data set, 33.82% (7591) sequences were annotated as OMP.nn by HHomp [14], which means there was no closely related homolog of known structure found for these proteins and thus, the number of β-strands in them is unknown. Thus, it is not possible to filter the dataset based on OMP class alone. But, as a control, we removed one OMP class at a time from the dataset and checked for differences in the clustering. When removing OMP.8 (Figure 9A) and OMP.12 (Figure 9B), two OMP classes that are not overrepresented in any of the taxonomy classes; this did not visibly affect the clustering. But when we removed the OMP.16 (Figure 9C) or the OMP.22 (Figure 9D) class, which have a high prevalence in β-proteobacteria and α-proteobacteria, respectively, this changed the clustering behavior of the respective taxonomic classes significantly; the organisms got scattered away from their position in the cluster compared to the situation in Figure 1A. This shows that the over-representation of certain OMP classes can influence the peptide sequence space, but since the proteins from over-represented OMP classes still contribute to the real sequence space of the organisms, we decided not to correct for this effect and used all peptides from the organisms in our experiments.

**Figure 10:**
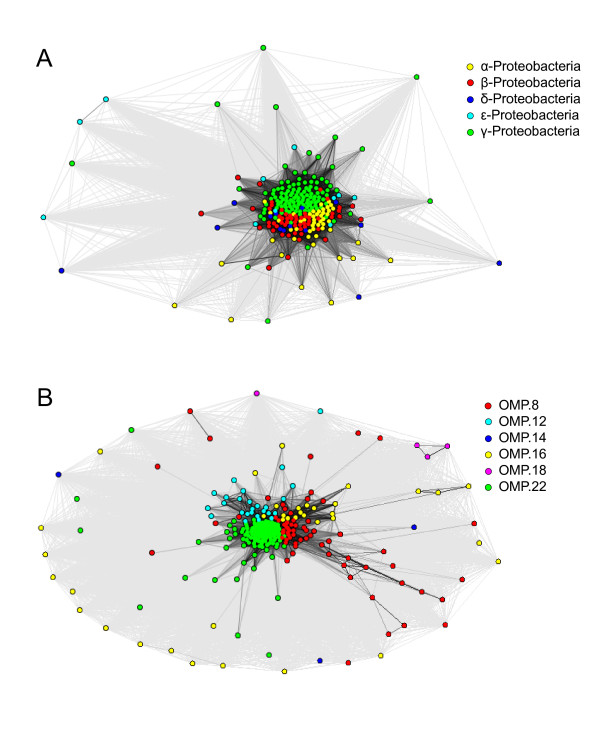
**CLANS cluster map of OMP-Organism class based entities.** In figure 10**A** and figure 10**B**, each node is a representative of OMP-Organism entities that have more than five unique peptides of a single OMP class from an individual organism. In Figure 10A, entities are only from the OMP.22 class, which includes entities from all proteobacterial taxonomic classes. In Figure 10B, entities are only from γ-Proteobacteria and include different OMP classes.

We also examined whether there is a more general signal from OMP classes, other than the signal from the over-representation of an individual OMP class that would influence the observed organism-specific signal. For this, we separated the peptides from an organism based on the OMP classification and selected the entities which had more than five unique peptides for further analysis. From this, we created two data sets of entities; one data set containing organisms from all taxonomic classes, but with C-terminal insertion signals only from 22-stranded OMPs, and a second data set containing organisms only from γ-proteobacteria, but in which individual organisms were split into multiple entities, each representing an OMP class that contained more than five unique C-terminal insertion signals. We clustered these data sets separately and the resulting cluster maps are shown in Figure 10A and B. In the cluster map in Figure 10A, each node is an organism, but only the C-terminal insertion signals from 22-stranded OMP class were considered for the clustering. In this cluster map, all the organisms clustered based on their taxonomic classes. In the cluster map in Figure 10B, all organisms are from γ-proteobacteria, but organisms with multiple OMP classes with more than five unique C-terminal insertion signals per class will result in multiple representative nodes. These nodes which belong to different OMP classes clustered based on the OMP classes. This confirms that there are independent contributions to the overall signal, from both the OMP classes and from taxonomy. Within one OMP class, there still is divergence in accordance with different taxonomic classes; but overrepresentation of a single OMP class in an organism influences the average motif of an organism.

## Conclusion

In our study, we were able to reproduce the difference between *E. coli* and *Neisseria* C-terminal β-strands as found by Robert *et al.,* which suggests a species-specific insertion signal for OMPs. But in contrast to the earlier report, we show that positively charged amino acids at the +2 position can not be the reason for the experimentally observed species specificity between these organisms, as *Escherichia* also contains C-terminal β-strands with positively charged amino acids at the +2 position. Moreover, there is experimental evidence which shows the functional expression of a heterologous OMP, YadA of *Yersinia enterocolitica,* with a positively charged amino acids at the +2 position, in *E. coli*[22]. The neisserial PorA protein and the neisserial C-terminal β-strands used by Robert *et al.* contained His at the +3 position, which is common for many OMP.16 proteins from β-proteobacteria and is not found in *Escherichia* OMPs; this might be the true difference in the recognition of C-terminal β-strands by the *Escherichia* BAM complex. Furthermore we found that *Helicobacter* strains form a distinct cluster in the cluster map, which is due to their very different composition of C-terminal β-strands. There is experimental evidence showing that expression of *H. pylori* OMPs in *E. coli* is lethal, and that this lethality can be suppressed by removing the C-terminal strand. When we looked at the frequency motifs from *Helicobacter* strains we did not notice a strong preference of any amino acid at the +2 or the +3 position, however we observed a strong preference of Tyr at the +5 position, which is not common in *Escherichia* or other Proteobacteria. We assume that this position may play an important role in the rejection of these C-terminal β-strands by the *E. coli* BAM complex. The examples of *Neisseria* and *Helicobacter* show that different positions in the C-terminal recognition motif can be relevant for heterologous expression of OMPs. We predict that in certain group of species the highly preferred residues in certain positions of the C-terminal insertion signals are responsible for the inadequate recognition of the C-terminal insertion signals by the *E. coli* BAM complex. In the future, mutation studies will have to be performed to prove the importance of these residues in the recognition step in the OMPs biogenesis.

As a result of our study, we have shown that there is a large overlap between the signals from C-terminal insertion peptides of different organisms, which suggests that in most cases, heterologous expression should be possible. OMPs can fold *in vitro* even without the help of any other proteins [25]. The BAM complex is an enzyme that makes the folding of OMPs into the outer membrane more efficient by increasing the reaction rate of a natural process. Enzymes modify reaction rates by changing the reaction route to lower the activation energy, and binding/recognition is part of this changed route. Thus, it is also important to consider expression rates: poor recognition might still lead to properly folded OMPs in the outer membrane of a heterologous host at low expression rates. But under overexpression conditions, the BAM machinery can probably not cope with poorly recognized signals that would lead to lower overall folding rates (considering that recognition is the first and probably in some cases rate-limiting step of the folding process). Different classes of OMPs have different folding rates, where small OMPs fold faster and more efficiently (again *in vitro*) than larger ones, which might explain why large OMPs seem to depend more heavily on an intact BAM machinery than small ones [26,27].

Since there are two different signals that contribute to the observed average motifs, from OMP class and from taxonomy, it is problematic to use averaged motifs or sequence logos to determine the compatibility of a given protein-organism pair. The main problem here is the overrepresentation of certain OMP classes in some organism groups; this overrepresentation shifts the average signals. It is more useful to determine for an individual C-terminal motif form a protein to be expressed, whether it is also present in any of the OMPs of the host organism.

The taxonomy-based specificity we observed here based on sequence space depends upon the entire peptide sequence, but at the functional level, these peptides are recognized based on the interacting residue positions in the C-terminal insertion signal peptide. The PDZ domain of the bacterial periplasmic stress sensor, DegS, also recognizes the C-terminal YxF motif in the last β strand of misfolded OMPs. This leads to the activation of the proteolytic pathway and the expression of DegP, which degrades misfolded OMPs [28,29]. Since the C-terminal β-strand is recognized by both the PDZ domain of the DegS protein and by the BAM complex, studying the co-evolution of interacting residues in both cases would help in understanding the divergence of the C-terminal β-strands between different Gram-negative bacterial organisms. Unfortunately, co-crystal structures of the BAM complex with its substrates are not available yet. With more experimental evidence about the substrate recognition sites for the C-terminal insertion signal peptide in the BAM complex, the co-evolution of the interacting amino acids can hopefully be studied in the future, which may shed more light on into the evolution of the BAM machinery in different Proteobacteria, and on its ability to recognize heterologous substrates for biotechnology applications.

## Methods

### Predicting outer membrane β-barrel proteins

In a previous study [30] to annotate the subcellular localizations (SCLs) for the proteomes of 607 Gram-negative bacteria, we developed the program/database ClubSub-P, in which we used programs like CELLO [13], PSORTb [12] and HHomp [14] to annotate OMPs. CELLO [13] and PSORTb [12] use support vector classifiers to annotate different SCLs of query sequences and are much faster than HHomp [14] which uses HMM-HMM-based search algorithms to predict and classify OMPs. Thus we used CELLO and PSORTb to scan all the sequences in the clusters of the ClubSub-P database. A random protein was selected from a cluster where CELLO or PSORTb had a positive hit for an outer membrane protein, and the sequence was analyzed with HHomp. When HHomp predicted a protein with more than 90% probability to be an OMP, we considered all the proteins in the cluster to be OMPs. We in addition selected all singleton sequences with positive prediction from CELLO or PSORTb and analyzed them with HHomp.

### Finding the C-terminal β-strands

HHomp annotates/classifies OMPs based on the number of β-stands present in them. HHomp calculates/predicts this from homologous structures of OMPs. We transferred this annotation from the best hit in HHomp runs to the query sequences. HHomp also annotates secondary structure and β-barrel strand predictions using PSIPRED [19] and ProfTMB [18], which was used to extract the C-terminal (last) β-strand/motif for each OMP. The last β-strand predicted by ProfTMB [18] was extracted as the C-terminal motif from representative sequences and singletons, and further filters were applied to reduce the false positive rate; 1) 70% of the amino acids in the motif should have a β-strand prediction from PSIPRED [19], 2) If the C-terminal of the protein is more than 4 residues away from the C-terminus of the motif, we extended the predicted motif by up to 4 amino acids to find an aromatic hydrophobic residue [F,Y,W], else we extended the C-terminus of the motif to the end of the protein itself. 3) Additionally, if the motif length was less than 10 residues, we extended the motif towards its N-terminus. 4) Furthermore with the regular expression.

[^C][YFWKLHVITMADGRE][^C][YFWKLHVITMADGRE][^C][YFWKLHVITMADGRE][^C].[^C][YFWHILM] (an updated version of BOMP [31] C-terminal pattern), we searched for the existence of the alternating hydrophobic pattern in the motif which is typical for transmembrane β-strands.

Using the information from this representative C-terminal motif, we extracted C-terminal motifs from the rest of the sequences in the clusters. We used MAFFT [32] to align the sequences from the cluster, and used the start and end coordinates of the C-terminal motif discovered above in the representative sequences randomly selected from the clusters. Motifs were extended on the both sides, in cases where we encountered gaps in the alignment. The gaps were removed and then resulting motifs were subjected to alternating hydrophobic pattern matching.

The peptides we collected vary in length from 10 to 21 residues (only six of the peptides were longer than 21). We then applied GLAM2 [33], a gapped motif discovery algorithm, to find the strongest motif with a length of 10 from this dataset. We found 24,626 motif instances in 25,454 sequences, and only 232 motifs in this alignment had gaps. The gapped motifs were removed before further analysis. 20,135 of the motif instances were C-terminal to the protein itself (which means there were no additional domains at the C-terminal end of the β-barrel proteins). 437 organisms had more than 20 unique C-terminal β-strands, ranging from 21 to 171 peptides in different organisms. In total, the 437 organisms yielded 22,447 peptides, of which 12,949 are unique peptides.

### Sequence based clustering

Since all of the peptides are 10 amino acids in length by default, we used the PAM30 substitution matrix for an all-against-all BLAST, with an E-value cut-off of 1000 and used the pairwise P-values to cluster the sequences in CLANS [20].

### PSSM profile-based hierarchical clustering

The relative frequencies of the 20 amino acids were calculated for all 10 positions in the peptides from an organism. To obtain odds scores, the relative frequencies were simply divided by each residue’s background frequency, which was calculated by shuffling the amino acid sequence in all the peptides from all organisms, and log base 2 was applied to obtain a PSSM matrix. The 20 x 10 PSSM matrices obtained for each organism were stored in a single 437x200 PSSM matrix, and correlation distances were calculated between each organism and agglomerative hierarchical clustering (average method) was performed via the pvclust [34], which calculates two types of *p*-values, AU (Approximately Unbiased) *p*-value and BP (Bootstrap Probability) value to indicate the likelihood of the cluster formation.

### Peptide sequence space-based clustering

#### Chemical descriptors

To generate a peptide sequence space, each amino acid in the peptide sequences was represented by five chemical descriptors that are the first five principal components derived from 26 physiochemical descriptor variables using dimensionality reduction techniques [35]. The initial 26 physiochemical descriptor variables include the molecular weight, experimentally determined retention values from seven thin-layer chromatography runs, van der Waals volume of the side chain, three nuclear magnetic resonance shift variables, log *P*, six variables for semiempirical molecular orbitals, three variables for total, polar and nonpolar surface area, two variables for side chain charge and two variables for hydrogen bond donor and acceptor [35]. The five principal components derived from these 26 variables contain the maximal variations in the data set and they can be interpreted as the size, polarizability, and the lipophilic, steric, and electronic properties of all the amino acids [35]. The amino acid descriptors were originally derived for use as design variables in peptide design, and in the construction of combinatorial libraries to effectively search chemical property space [35]. Here we used them to describe the space occupied by the C-terminal β-strands and to measure how strongly peptide sequences of different organisms overlap. Using the chemical descriptors, each amino acid in the peptide was converted into a 5-dimensional vector; thereby, each 10aa peptide was represented as a 50-dimensional vector. Thus, the whole set of 22,447 peptides were converted to a 22,447 x 50 matrix.

#### Principal component analysis

Since the dimensionality of the data set (50) is larger than the sample size (minimum 21 peptides per organism), the dimensionality of the peptide vectors had to be reduced below the sample size (i.e., below 21 in our dataset) for further statistical analysis [36]. Principal component analysis (PCA) is a mathematical technique to reduce the dimensionality of data sets, while retaining most of the variation in the data set. This is achieved by projecting the original data vectors along the directions of maximal variation, called principal components (PCs). The first PC captures the maximum variation; the variation associated with consecutive PCs decreases rapidly. Thus, the original data set can be mapped into a lower dimensional space by projecting the original data on those PCs representing most of the variation [36,37]. We used PCA to reduce the dimensionality of our peptide sequences (22,447 x 50 matrix) by projecting the 50 dimensional chemical descriptor vectors onto the first 12 principal components, which represent 69.05% of the total variation in the data. We thereby obtained a 22,447 x 12 matrix that did not suffer from any problems in sample size.

#### Multivariate Gaussian fitting and Hellinger distance

Next, we fit a multivariate Gaussian distribution for each individual organism by calculating a 12-dimensional mean vector and covariance matrix, (e.g., for *E. coli* 536 which has 66 unique peptides, the Gaussian will be fitted based on a 66 x 12 matrix).

**Figure 11:**
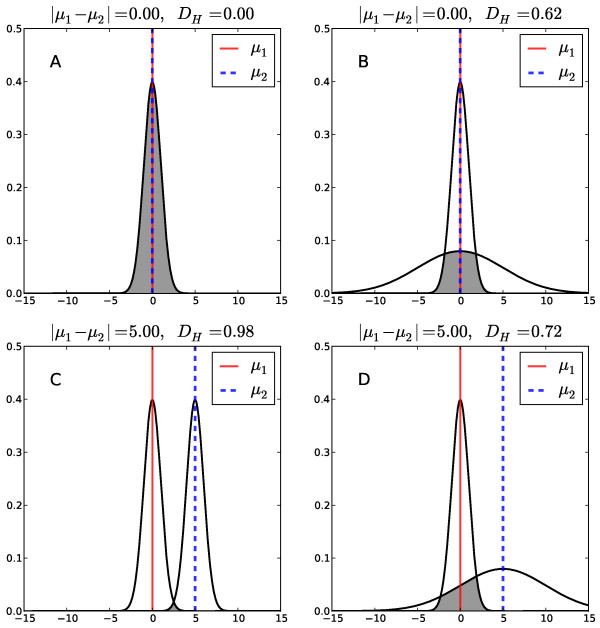
**Illustration of the difference between the Euclidean distance and the Hellinger distance for one-dimensional Gaussian distributions.** Two Gaussian distributions are shown as black lines for different choices of μ and σ. The grey area indicates the overlap between both distributions. |μ1−μ2| is the Euclidean distance between the centers of the Gaussians, D_H_ is the Hellinger distance (equation 1). Both values are indicated in the title of panels A-D. **A:** For μ1 = μ2 = 0, σ1 = σ2 = 1, the Euclidean distance and the Hellinger distance are both zero. **B:** For μ1 = μ2 = 0, σ1 =1, σ2 = 5 the Euclidean distance is zero, whereas the Hellinger distance is larger than zero because the distributions do not overlap perfectly (the second Gaussian is wider than the first). **C:** For μ1 =0, μ2 = 5, σ1 = σ2 = 1, the Euclidean distance is five, whereas the Hellinger distance almost attains its maximum because the distributions only overlap little. **D:** For μ1 =0, μ2 = 5, σ1 =1, σ2 =5, the Euclidean distance is still five as in C because the means did not change. However, the Hellinger distance is larger than in C because the second Gaussian is wider, which leads to a larger overlap between the distributions.

The Euclidean distance between means of peptide sequence spaces is not suitable for measuring the similarity between the C-terminal β-strands of different organisms. Instead, the similarity measure should also represent how strongly their associated sequence spaces overlap. To achieve this we used the Hellinger distance between the fitted Gaussian distributions [38]. In statistical theory, the Hellinger distance measures the similarity between two probability distribution functions, by calculating the overlap between the distributions. For a better understanding, Figure 11 illustrates the difference between the Euclidean distance and the Hellinger distance for one-dimensional Gaussian distributions. The Hellinger distance, D_H_(Org_1_,Org_2_), between two distributions Org1(x) and Org2(x) is symmetric and falls between 0 and 1. D_H_(Org_1_, Org_2_) is 0 when both distributions are identical; it is 1 if the distributions do not overlap [39]. Therefore we have for the squared Hellinger distance D_H_^2^(Org_1_, Org_2_) = 1 – overlap(Org1,Org2). The following equation (1) was derived to calculate the pairwise Hellinger distance between the multivariate Gaussian distributions, Org_1_ and Org_2_, where μ_1_ and μ_2_ are the mean vectors and Σ_1_ and Σ_2_ are the covariance matrices of Org_1_ and Org_2_, and *d* is the dimension of the sequence space, i.e. *d*=12

(1) $$\;D_{H}\left(Org_{1,}Org_{2}\right)=\sqrt{1-2^{d/2}\left(\frac{\det\left(\sum_{1}\right)\det\left(\sum_{2}\right)}{\det{\left(\sum_{1}\;+\sum_{2}\right)}^{2}}\right){\;}^{1/4}\;\text{exp}\;\left\{-\frac{1}{4}\;{\left(\mu_{1}-\mu_{2}\right)}^{T\;}{\left(\sum_{1}\;+\sum_{2}\right)}^{-1}\;\left(\mu_{1}-\mu_{2}\right)\right\}}$$

#### CLANS

Next, the Hellinger distance was used to define a dissimilarity matrix for all pairs of organisms. The dissimilarity matrix was converted to P-values, which were then used as input in CLANS [20] to compute a cluster map showing all organisms. CLANS is a graph-based clustering method that represents sequences as nodes. All nodes are connected by weighted edges where the pairwise similarity between the sequences determines the strength of the weight [20]. In our study, individual organisms were considered as nodes and the weight of the edges connecting the nodes was based on the pairwise Hellinger distance (pairwise overlap of sequence space) between the organisms. Hence stronger connections represent a larger overlap/similarity between the peptide sequence spaces, while organisms with high divergence in their C-terminal motifs are only weakly connected or completely disconnected in the cluster map. Initially the nodes are randomly placed in a 2D space and experience attraction forces according to how strongly they are connected with the other nodes. In an iterative refinement scheme, nodes move towards similar nodes with an attractive force proportional to the similarity between them. A small, overall repulsive force is applied to all pairs of nodes to keep them from collapsing into a single node. Since CLANS [20] uses non-deterministic dynamics, each run performed with the same dataset will result in a similar but not necessarily identical clustering. Thus, multiple clustering runs were performed to check the reproducibility of the final clustering. Because initial tests showed that with the default attraction and repulsion values nodes (organisms) were collapsing, we used very small attraction values (up to 0.1) and high repulsion values (up to 500) to avoid collapse of nodes and to obtain visually better clusters.

### Frequency plot

The WebLogo [40] online tool was used to create the frequency plots, using custom colors. Only unique peptide sequences were used to generate all the frequency plots. The amino acid percentage plots were created using R version 2.13.1 [41].

## Competing interests

There is no competing interest.

## Authors’ contributions

NP generated and analyzed the data. MH provided the initial script for pairwise Hellinger distance calculation. DL conceived the initial idea about the project and helped in drafting the manuscript. NP wrote the manuscript, MH and DL read and improved the manuscript. All authors approved the manuscript.

## Supplementary Material

Additional file 1 The figure shows the number the over representation of OMP.16 proteins among β-proteobacteria and OMP.22 among α-proteobacteria. Click here for file

Additional file 2 The table lists the number of OMPs in an organism present in different OMP classes. Click here for file
