# Supplementary figures and images for: Is the C-terminal insertional signal in Gram-negative bacterial outer membrane proteins species-specific or not?
Source: BMC Genomics. 2012 Sep 26;13:510. doi: 10.1186/1471-2164-13-510 (PMC3582582; doi:10.1186/1471-2164-13-510)

Number of proteins in each OMP class

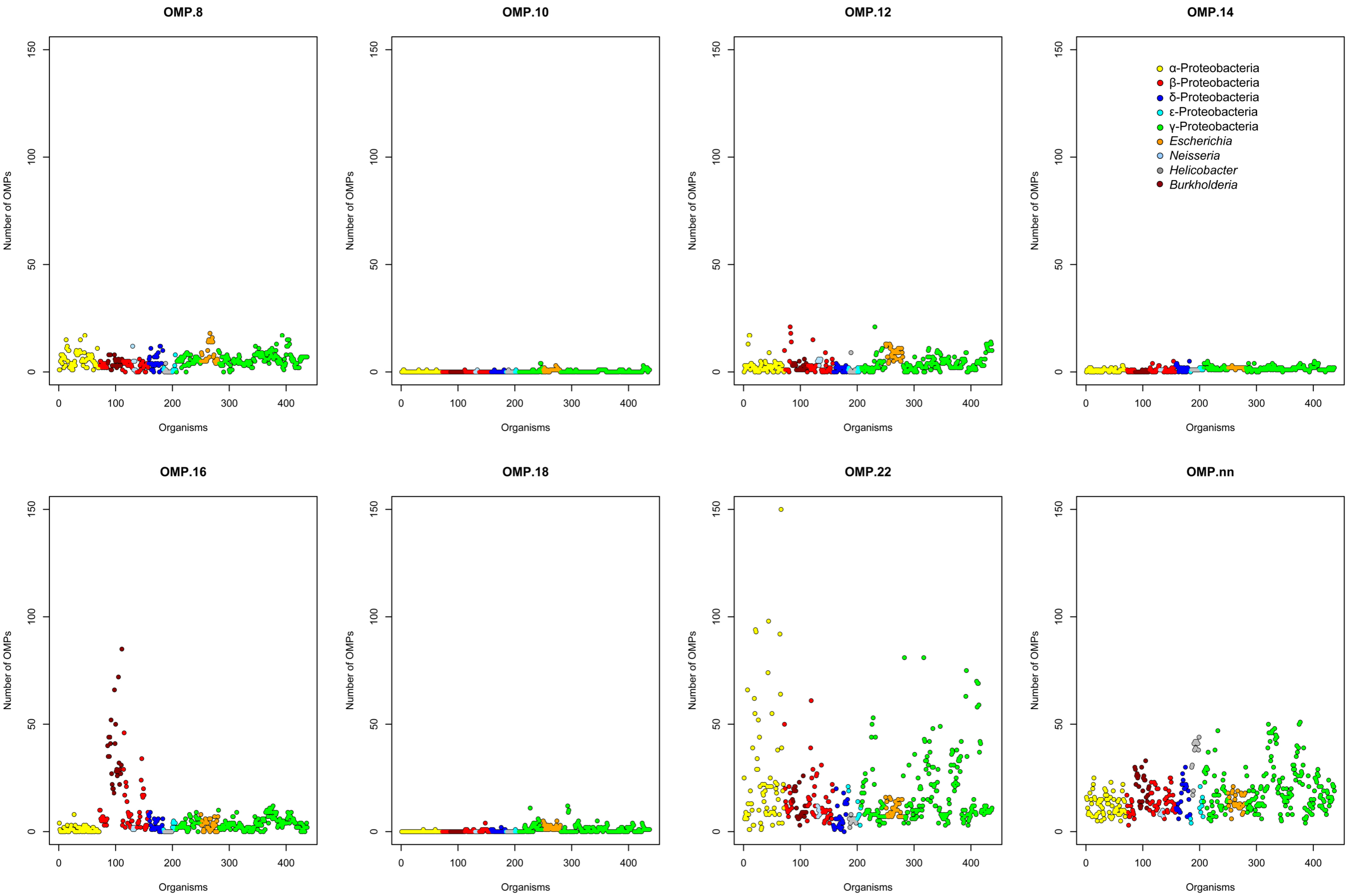

Supplement: Additional file 1 — The figure shows the number the over representation of OMP.16 proteins among β-proteobacteria and OMP.22 among α-proteobacteria. [file 1471-2164-13-510-S1.pdf]
